# Serum uric acid to creatinine ratio as marker of early vascular damage and renal tubular injury in non-albuminuric diabetic kidney disease

**DOI:** 10.1007/s40618-026-02972-7

**Published:** 2026-07-10

**Authors:** Maurizio Di Marco, Sabrina Scilletta, Nicoletta Miano, Nicola Marrano, Annalisa Natalicchio, Francesco Giorgino, Giosiana Bosco, Francesco Di Giacomo Barbagallo, Roberto Scicali, Andrea Tumminia, Agostino Milluzzo, Lucia Frittitta, Salvatore Piro, Antonino Di Pino

**Affiliations:** 1 Department of Clinical and Experimental Medicine, Internal Medicine, University of Catania, Garibaldi Hospital, Via Palermo, 636 95122 Catania, Italy; 2 Department of Precision and Regenerative Medicine and Ionian Area, Section of Internal Medicine, Endocrinology, Andrology and Metabolic Diseases, University of Bari Aldo Moro, 70124 Bari, Italy; 3 Department of Medicine and Surgery, “Kore” University of Enna, 94100 Enna, Italy; 4 Endocrine Unit, Garibaldi-Nesima Hospital, Catania, Italy

**Keywords:** Type 2 diabetes, Non-albuminuric diabetic kidney disease, Serum uric acid to creatinine ratio, Arterial stiffness, Cardiovascular risk, Tubular damage

## Abstract

**Purpose:**

Serum uric acid to creatinine ratio (SUA/sCr) has emerged as potential biomarker for non-albuminuric diabetic kidney disease (NA-DKD), a recently recognized high-prevalence DKD phenotype. However, the relationship between SUA/sCr and cardiovascular and renal injury profile in this population is not well established. This study aimed to evaluate SUA/sCr across the spectrum of DKD, particularly focusing on NA-DKD, and to test its association with subclinical vascular damage, urinary biomarkers and ultrasound features of kidney damage.

**Methods:**

Presence of carotid plaques, pulse wave velocity (PWV), renal resistive index (RRI), and urinary biomarkers of tubular injury were assessed in 207 individuals with type 2 diabetes. Participants were split based on estimated-glomerular-filtration-rate (eGFR) and urinary-albumin-to-creatinine-ratio (UACR) into four groups: controls (UACR < 30 mg/g, eGFR ≥ 60 ml/min/1.73m^2^), A-DKD (Albuminuric-DKD; UACR ≥ 30 mg/g, eGFR ≥ 60 ml/min/1.73m^2^), NA-DKD (Non-albuminuric-DKD; UACR < 30 mg/g, eGFR < 60 ml/min/1.73m^2^), A&L-DKD (Albuminuric-and-Low-eGFR-DKD; UACR ≥ 30 mg/g, eGFR < 60 ml/min/1.73m^2^).

**Results:**

Participants with NA-DKD showed a lower SUA/sCr than those with A-DKD and controls (4.71 ± 1.52 vs 6.06 ± 1.70 vs 6.67 ± 1.87, both *P* < 0.0001). A lower SUA/sCr was independently correlated with NA-DKD (β = -1.63, *P* < 0.0001) and A&L-DKD (β = -2.01, *P* < 0.0001). SUA/sCr was inversely and independently associated with urinary β2-microglobulin (β = -0.21, *P* = 0.0082). Moreover, lower SUA/sCr was associated with PWV > 10 m/s (OR 0.77, 95%CI 0.63–0.95, *P* = 0.014), the presence of carotid plaques (OR 0.78, 95%CI 0.63–0.96, *P* = 0.020), and higher RRI (β = -0.18, *P* = 0.0082).

**Conclusion:**

In T2D, lower SUA/sCr correlated with NA-DKD, but not with A-DKD. Lower SUA/sCr was associated with subclinical vascular and tubular damage. Future studies are needed to test SUA/sCr as candidate biomarker to improve DKD risk stratification.

## Introduction

Diabetic kidney disease (DKD) is a frequent complication of type 2 diabetes (T2D) with a potential severe clinical impact, representing one of the leading causes of end-stage renal disease and haemodialysis [1, 2]; however, it is becoming increasingly clear that DKD is a highly heterogeneous clinical entity. In recent years, a specific phenotype characterized by reduced estimated glomerular filtration rate (eGFR) without altered urinary albumin-to-creatinine ratio (UACR), known as non-albuminuric DKD (NA-DKD), has been described. This phenotype has significant clinical impact due to its increasing prevalence [3, 4].

NA-DKD presents a clinical challenge in early diagnosis due to its atypical presentation, and data on the differential cardiovascular risk and renal damage profile of this phenotype in comparison with the classical albuminuric DKD (A-DKD) is still debated [5, 6]. In a recent study, we highlighted a worse cardiovascular and renal injury profile in individuals with NA-DKD in comparison to A-DKD, emphasizing the central role of eGFR in the risk stratification of people living with diabetes [7]. However, there is increasing interest in identifying additional simple and cost-effective biomarkers, that should capture differences in cardiovascular risk, renal glomerular and/or tubular dysfunction [8–11].

Alongside the well-established role of serum uric acid as a biomarker of cardiovascular risk, there has been growing interest over the past few years in using the serum uric acid to serum creatinine ratio (SUA/sCr), as a diagnostic tool for DKD [12]. To date, a low SUA/sCr has been shown to be a predictor of adverse outcomes—such as cardiovascular events, mortality, and stroke recurrence—across different clinical contexts [13–15]. Furthermore, Mu H. et have recently described SUA/sCr as a significant and independent predictor of NA-DKD in a large cohort of patients with T2D [12]. However, the relationship between this index and the cardiovascular and renal injury profile in this population has not been fully investigated.

The aim of the present study was to assess SUA/sCr across the spectrum of different phenotypes of DKD and to test its association with subclinical vascular damage in terms of arterial stiffness and presence of carotid atherosclerotic plaques, as well as with urinary biomarkers and ultrasound features of kidney damage. In consideration of the increasing prevalence, we particularly focused our attention on patients with NA-DKD.

## Methods

### Study subjects

A total of 207 patients with type 2 diabetes, who attended our department for diabetes and cardiovascular risk evaluation at Garibaldi Nesima Hospital, Catania, Italy, were recruited in this study. The inclusion criterion was an age range 45–75 years. People with autoimmune diseases as well as under corticosteroids therapy, with malignancies, therapy with allopurinol or febuxostat, and history of drug abuse were excluded. All participants underwent a complete physical examination, as well as a comprehensive review of clinical history, smoking status, and medications.

Body mass index (BMI) was calculated as weight (kg)/[height (m)]^2^. Blood pressure (BP) was measured after 10 min resting using a calibrated sphygmomanometer. Venous blood samples were drawn from the antecubital vein in the morning in fasting status. Low density lipoprotein (LDL) cholesterol concentrations were estimated using the Friedewald formula. A sample of spot urine was collected.

### Biochemical analysis

Plasma glucose, serum total cholesterol, triglycerides, and HDL cholesterol were measured using available enzymatic methods [16]. Glycated haemoglobin (HbA_1c_) was measured via high-performance liquid chromatography using a National Glycohemoglobin Standardization Program and was standardized to the Diabetes Control and Complications Trial (DCCT) assay reference [17, 18]. Chromatography was performed using a certified automated analyser (HLC-723G7 haemoglobin HPLC analyser; Tosoh Corp.) (normal range 4.25–5.9%).

eGFR was assessed with the Chronic Kidney Disease Epidemiology Collaboration (CKD-EPI) equation [19] and albuminuria was determined as mean values of two measurements in a period of 3–6 months of UACR in spot urine samples [20].

### Study groups

Participants, taking into account UACR and eGFR, were assigned to four different groups: controls (UACR < 30 mg/g and eGFR ≥ 60 ml/min/1.73m^2^), A-DKD (UACR ≥ 30 mg/g and eGFR ≥ 60 ml/min/1.73m^2^), NA-DKD (UACR < 30 mg/g and eGFR < 60 ml/min/1.73m^2^), A&L-DKD (Albuminuric and Low eGFR DKD; UACR ≥ 30 mg/g and eGFR < 60 ml/min/1.73m^2^).

### Carotids ultrasound examinations

All ultrasound examinations were performed by a single physician who was blinded to the clinical and laboratory characteristics of the patients, using a high-resolution B-mode ultrasound system. Longitudinal B-mode (60 Hz, 128 radiofrequency lines) images of the common carotid arteries 2 cm below the carotid bulb were obtained using a high-precision echo tracking device (MyLab Alpha, Esaote, Maastricht, NL) paired with a high-resolution linear array transducer (13 MHz) to assess the presence of atherosclerotic plaques and to acquire intima-media thickness (IMT) as previously described [21]. The mean value of three measurements for each carotid was considered.

### Pulse wave velocity

Pulse wave velocity (PWV) was determined by SphygmoCor CvMS (AtCor Medical, Sydney, Australia) system, that uses a tonometer, and two different pressure waves obtained at the common carotid artery (proximal recording site) and at the femoral artery (distal recording site). The start of the pulse wave was identified using a concomitant electrocardiogram registration. The PWV was computed as the difference in travel time of the pulse wave between the two different recording sites and the heart, divided by the travel distance of the pulse waveform. The PWV was calculated on the mean basis of 10 consecutive pressure waveforms to cover a complete respiratory cycle as previously described [22].

### Renal resistive index (RRI)

The Doppler spectrum of intrarenal arteries was used to derive renal resistive index (RRI) as the difference between maximum (peak systolic) and minimum (end-diastolic) flow velocity to maximum flow velocity. The measure was performed using a convex transducer connected to a high-precision echo tracking device (MyLab Alpha, Esaote, Maastricht, NL) [23]. The mean value of 3 different measurements was considered.

### Quantification of urinary renal function biomarkers

The BioPlex® (Bio-Rad, Hercules, California, USA) platform was used for the determination of urinary biomarkers of kidney damage as previously described [7]. The commercial package Bio-Plex Pro™ RBM Human Kidney Toxicity Assay Panel 2 (urinary albumin, urinary β2-microglobulin [u-B2M], urinary cystatin C [u-Cys], urinary Neutrophil Gelatinase-Associated Lipocalin [u-NGAL], urinary osteopontin [u-OPN], and urinary TreFoil Factor 3 [u-TFF3]) was used to evaluate the protein content in the urinary samples, according to the manufacturer’s protocol.

### Statistical analysis

The sample size was calculated based on SUA/sCr ratio using a level of significance (α) set to 5% and a power (1-β) set to 80%. According to the study of Mu et al. [12], a mean difference of 0.72 ± 0.92 in the SUA/sCr between NA-DKD group and controls was considered. The estimated sample size was 25 patients per group.

Statistical comparisons of clinical and biomedical parameters were performed using Stat View 6.0 for Windows. The data are presented as mean ± SD, median (1st – 3rd quartile), or absolute number (percentage) as appropriate. The distributional characteristics of variables were assessed using the Kolmogorov–Smirnov test. When necessary, numerical variables were logarithmically transformed to reduce skewness. The comparison between study groups was performed with one-way ANOVA for continuous variables and χ^2^ test for categorical variables. The Bonferroni post hoc test for multiple comparisons was also performed. A *P* value < 0.05 was considered significant.

To test the correlation between SUA/sCr and the allocation to study groups, a multiple regression analysis, adjusting for age, sex, BMI, history of hypertension, and use of sodium-glucose cotransporter 2 inhibitors (SGLT2i) was performed. Furthermore, to identify the major determinants of SUA/sCr three multivariable regression models were fitted: the first included age, sex, BMI, and systolic BP (SBP), and therapy with SGLT2i; variables reaching significance in the first model were included in a second model including biochemical variables (LDL and HDL cholesterol, HbA1c, UACR > 30 mg/g); finally, variables reaching significance in the second model were included in a third model with renal injury biomarkers (u-B2M, u-Cys, u-NGAL, u-OPN, and u-TFF3).

To identify variables independently associated with variations of RRI, we performed three multivariate regression models as well: the first two models were the same as above, while the third model incorporated SUA/sCr. The same models were used to perform a multiple logistic regression analysis to identify variables independently associated with the presence of atherosclerotic plaques, and with having altered PWV, with a cut-off value of 10 m/s [24].

The variance inflation factor (VIF) was used to check for multicollinearity among the predictor variables in multiple regression analysis. Any variable with a VIF that exceeded 4 was excluded from the model. No variable was detected with a VIF greater than 4.

The study was in accordance with the Declaration of Helsinki and was approved by the local ethics committee (Comitato Etico Catania 2, protocol n. 270/C.E. 26 April 2022). Informed consent was obtained from each participant.

## Results

### General characteristics of the study population

According to the eligibility criteria, 207 participants with type 2 diabetes were included in this study. The study population was divided into the following four groups (based on UACR and eGFR levels): 62 subjects without DKD (Controls), 54 subjects with albuminuria (A-DKD), 47 subjects with eGFR reduction without albuminuria (NA-DKD), and 44 subjects with both albuminuria and eGFR reduction (A&L-DKD). As shown in Table 1, the study population showed a higher prevalence of men, which was homogenous in all the study groups. Age was significantly higher in NA-DKD and A&L-DKD participants in comparison with the others. The study groups were homogeneous for the percentages of patients with history of cardiovascular disease (CVD) (myocardial infarction, stroke and peripheral artery disease), except for the A&L-DKD group, which showed a significantly higher percentage of subjects with a history of CVD in comparison with all the other groups. The lipid profile was comparable among study groups, apart from TG, that were higher in A&L-DKD. Regarding medications, participants with NA-DKD and A&L-DKD showed a lower use of metformin and higher use of SGLT2i, as expected.

**Table 1:** Clinical and metabolic characteristics of the study population according to UACR and eGFR

	Controls (n = 62)	A-DKD (n = 54)	NA-DKD (n = 47)	A&L-DKD (n = 44)
Age, years	63.89 ± 7.15	63.02 ± 6.80	68.68 ± 6.01^*†^	69.66 ± 5.43^*†^
Sex, no. (%) of females	20 (32.26)	9 (16.67)	14 (29.79)	10 (22.72)
Active smokers, no. (%)	15 (24.19)	29 (53.70)^*^	9 (19.14)^†^	12 (27.27)^†^
History of CVD^§^, no. (%)	14 (22.58)	14 (25.92)	6 (12.76)	23 (52.27)^*†‡^
BMI, kg/m^2^	29.05 ± 5.22	30.00 ± 5.14	27.64 ± 3.11^†^	27.68 ± 4.20^†^
SBP, mmHg	130.92 ± 15.25	139.71 ± 17.39^*^	133.78 ± 10.98	133.57 ± 15.35
DBP, mmHg	76.42 ± 9.88	82.12 ± 11.65^*^	78.78 ± 8.27	76.43 ± 9.77^†^
HbA_1c_, %	7.21 ± 1.21	7.60 ± 1.62	6.85 ± 0.97^†^	7.06 ± 1.21^†^
Fasting glucose, mg/dL	135.39 ± 38.02	140.43 ± 49.32	130.64 ± 48.91	124.02 ± 31.76
eGFR, mL/min/1,73m^2^	91.73 ± 12.77	90.82 ± 11.00	50.72 ± 8.28^*†^	48.58 ± 13.29^*†^
UACR, mg/g	11.00 (9.00 – 16.00)	71.50 (42.00 – 224.00)^*^	11.00 (7.00 – 17.00)^†^	120.50 (55.00 – 385.00)^*†‡^
Uric acid, mg/dl	5.00 ± 1.35	4.92 ± 1.30	6.24 ± 1.62^*†^	5.85 ± 1.42^*†^
SUA/sCr	6.67 ± 1.87	6.06 ± 1.70^*^	4.71 ± 1.52^*†^	4.25 ± 1.40^*†^
Total cholesterol, mg/dl	153.92 ± 39.53	149.20 ± 40.66	146.57 ± 33.37	148.75 ± 32.90
HDL cholesterol, mg/dl	46.77 ± 11.66	44.59 ± 11.66	45.48 ± 11.07	46.36 ± 11.63
LDL cholesterol, mg/dl	81.50 ± 32.40	76.31 ± 30.99	75.09 ± 32.03	70.69 ± 32.85
Triglycerides, mg/dl	110.00 (81.00 – 150.00)	104.50 (81.00 – 150.00)	102.50 (76.00 – 149.00)	138.50 (109.50 – 189.50)^‡^
MEDICATIONS				
Metformin, no. (%)	60 (96.77)	48 (88.89)	27 (57.45)^*†^	27 (61.36)^*†^
Insulin, no. (%)	19 (30.64)	19 (35.18)	13 (28.26)	23 (52.27)^*‡^
SGLT2 inhibitors, no. (%)	13 (20.97)	21 (38.89)	20 (42.55)^*^	25 (56.82)^*^
DPP4 inhibitors, no. (%)	9 (14.52)	2 (3.70)	8 (17.02)	3 (6.82)
GLP1-RAs, no. (%)	25 (40.32)	21 (38.89)	23 (48.94)	17 (38.64)
Sulphonylureas, no. (%)	2 (3.23)	2 (3.70)	2 (4.25)	−
Antithrombotics, no. (%)	35 (56.45)	28 (51.85)	23 (48.94)	25 (56.82)
Statins, no. (%)	44 (70.97)	43 (79.63)	34 (72.34)	35 (79.54)
ACE-i/ARBs, no. (%)	38 (61.29)	39 (72.2)	35 (74.47)	29 (65.91)

Data are presented as mean ± SD, median (IQR), or percentage. A-DKD: albuminuric diabetic kidney disease (UACR ≥ 30 mg/g and eGFR ≥ 60 ml/min/1.73 m^2^); NA-DKD: non-albuminuric diabetic kidney disease (UACR < 30 mg/g and eGFR < 60 ml/min/1.73 m^2^); A&L-DKD: albuminuric and low estimated glomerular filtration rate diabetic kidney disease (UACR ≥ 30 mg/g and eGFR < 60 ml/min/1.73 m^2^); CVD: cardiovascular disease; BMI: body mass index; SBP: systolic blood pressure; DBP: diastolic blood pressure; eGFR: estimated glomerular filtration rate; UACR: urinary albumin to creatinine ratio; SUA/sCR: serum uric acid to serum creatinine ratio; SGLT2: sodium-glucose transporter 2; DPP4: dipeptidyl-peptidase 4; GLP1-RAs: glucagon like peptide 1 – receptor agonists. ACE-i: angiotensin converting enzyme inhibitors; ARBs: Angiotensin receptor blockers. ^§^history of CVD includes myocardial infarction, stroke, and peripheral artery disease. ^*^P < 0.05 vs group A; ^†^ *P* < 0.05 vs group B; ^‡^ *P* < 0.05 vs group C

### SUA/sCr in the study population

Participants with NA-DKD showed a lower SUA/sCr than those with A-DKD and controls (4.71 ± 1.52 vs 6.06 ± 1.70 vs 6.67 ± 1.87, both *P* < 0.0001) (Table 1). Furthermore, NA-DKD did not show any significant difference in SUA/sCr ratio with A&L-DKD (4.71 ± 1.52 vs 4.25 ± 1.40, *P* = 0.19).

**Fig. 1:**
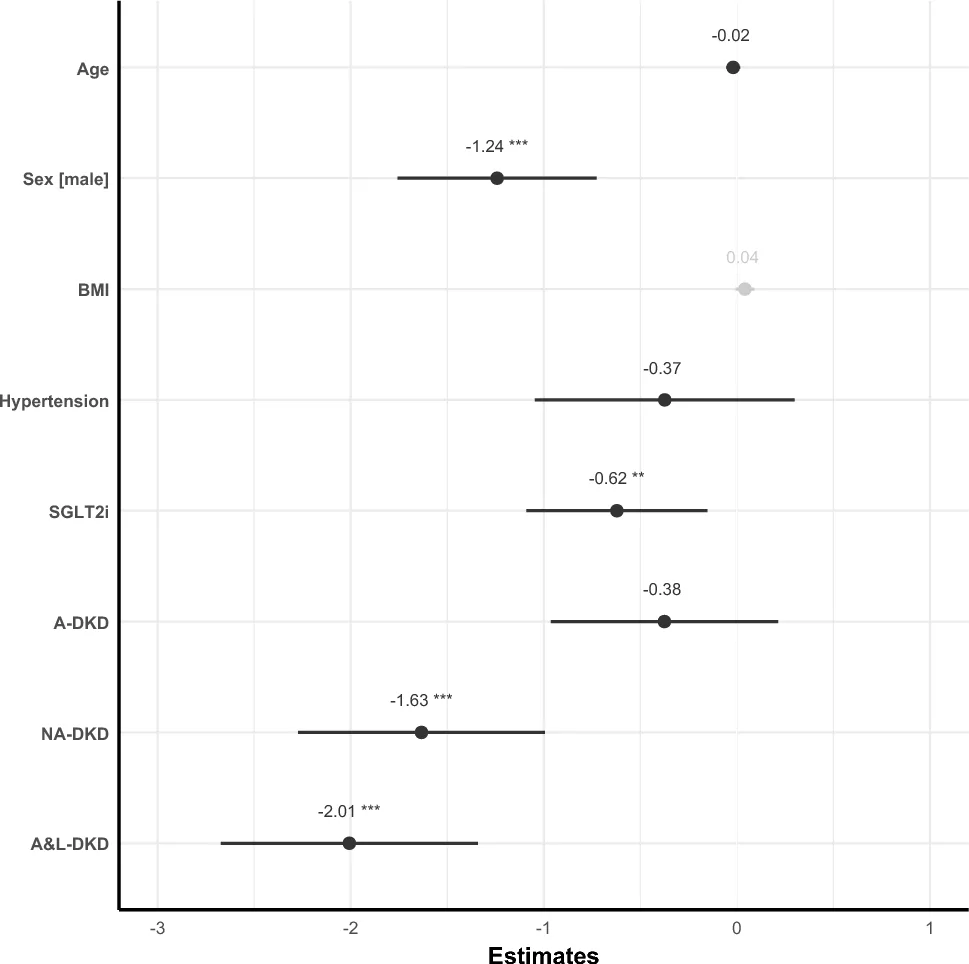
Multiple regression model analysing the correlation between SUA/sCr and DKD phenotypes. The graph shows the estimates of serum uric acid to serum creatinine ratio (SUA/sCr) in a multiple regression model, with age, sex, BMI, hypertension, therapy with sodium-glucose cotransporter 2 inhibitors (SGLT2i), and diabetic kidney disease (DKD) phenotypes as covariates. BMI: body mass index. A-DKD: albuminuric diabetic kidney disease (UACR ≥ 30 mg/g and eGFR ≥ 60 ml/min/1.73 m^2^); NA-DKD: non-albuminuric diabetic kidney disease (UACR < 30 mg/g and eGFR < 60 ml/min/1.73 m^2^); A&L-DKD: albuminuric and low estimated glomerular filtration rate diabetic kidney disease (UACR ≥ 30 mg/g and eGFR < 60 ml/min/1.73m^2^). * *P* < 0.05; ** *P* < 0.001; *** *P* < 0.0001

SUA/sCr remained significantly lower in NA-DKD (β = -1.63, *P* < 0.0001) and A&L-DKD (β = -2.01, *P* < 0.0001) even after adjusting for age, sex, BMI, history of hypertension, and therapy with SGLT2i (Fig. 1). Furthermore, sex did not significantly interact with the association of SUA/sCr with NA-DKD (*P* for interaction = 0.52) and A&L-DKD (*P* for interaction = 0.76).

### Cardiovascular profile of the study population according to UACR and eGFR

As shown in Table 2, participants with NA-DKD showed a higher PWV (11.56 ± 3.91 vs 9.73 ± 3.39 m/s,* P* = 0.029) in comparison to controls, as well as higher IMT (0.88 ± 0.18 vs 0.82 ± 0.14, *P* = 0.049). As regards carotid atherosclerotic plaque, A&L-DKD group had a higher prevalence than controls (*P* = 0.020). The NA-DKD group did not show any significant differences for the above-mentioned parameters in comparison to A&L-DKD group.

**Table 2:** Cardiovascular and renal profile of the study population according to UACR and eGFR

	Controls (n = 62)	A-DKD (n = 54)	NA-DKD (n = 47)	A&L-DKD (n = 44)
PWV, m/s	9.73 ± 3.39	10.38 ± 3.45	11.56 ± 3.91^*^	12.40 ± 4.48^*†^
Carotid plaques, no. (%)	27 (43.55)	27 (50.00)	28 (59.57)	32 (72.73)^*^
IMT, mm	0.82 ± 0.14	0.83 ± 0.16	0.88 ± 0.18^*^	0.88 ± 0.15
RRI	0.71 ± 0.08	0.70 ± 0.08	0.76 ± 0.10^*†^	0.78 ± 0.08^*†^
u-B2M, ng/ml	13.83 (4.18 – 33.41)	20.18 (6.20 – 66.69)	13.00 (5.89 – 61.22)	55.42 (8.62 –381.15)^*†‡^
u-Cys, ng/ml	15.33 (8.52 – 29.18)	22.11 (10.94 – 27.93)	12.35 (7.54 – 19.60)	18.45 (11.5 – 43.17)^‡^
u-NGAL, ng/ml	18.67 (9.85 – 39.79)	16.22 (9.85 – 25.92)	16.12 (10.16 – 30.71)	22.29 (14.45 – 45.40)
u-OPN, μg/ml	0.60 (0.39 – 0.83)	0.75 (0.42 – 1.01)	0.57 (0.34 – 0.96)	0.82 (0.51 – 1.31)
u-TFF3, μg/ml	1.25 ± 0.68	1.34 ± 0.65	1.58 ± 0.89^*^	1.37 ± 0.66

Data are presented as mean ± SD or percentage. A-DKD: albuminuric diabetic kidney disease (UACR ≥ 30 mg/g and eGFR ≥ 60 ml/min/1.73 m^2^); NA-DKD: non-albuminuric diabetic kidney disease (UACR < 30 mg/g and eGFR < 60 ml/min/1.73 m^2^); A&L-DKD: albuminuric and low estimated glomerular filtration rate diabetic kidney disease (UACR ≥ 30 mg/g and eGFR < 60 ml/min/1.73 m^2^); UACR: urinary albumin to creatine ratio; eGFR: estimated glomerular filtration rate; PWV: pulse wave velocity; AugI: augmentation index; qIMT: quality intima-media thickness; RRI: renal resistivity index; u-B2M: urinary β2 microglobulin; u-Cys: urinary Cystatin C; u-NGAL: urinary Neutrophil Gelatinase-Associated Lipocalin; u-OPN: urinary osteopontin; u-TFF3: urinary TreFoil Factor 3. ^*^ P < 0.05 vs group A; ^†^ *P* < 0.05 vs group B; ^‡^ *P* < 0.05 vs group C

### Renal profile of the study population according to UACR and eGFR

The RRI was significantly increased in individuals with NA-DKD compared with controls (0.76 ± 0.10 vs 0.71 ± 0.08,* P* = 0.016) and A-DKD group (0.76 ± 0.10 vs 0.70 ± 0.08,* P* = 0.0010). Moreover, we found no difference between NA-DKD and A&L-DKD groups (0.7 ± 0.10 vs 0.78 ± 0.08, *P* = 0.41) (Table 2).

As concerns urinary biomarkers of kidney injury (Table 2), participants with A&L-DKD (55.42 [8.62 – 381.15] ng/ml) showed higher levels of u-B2M in comparison to all the other study groups (controls: 13.83 [4.18 – 33.41]; A-DKD: 20.18 [6.20 – 66.69]; NA-DKD: 13.00 [5.89 – 61.22] ng/ml; All *P* < 0.050) and individuals with NA-DKD had higher levels of u-TFF3 in comparison to controls (1.58 ± 0.89 vs 1.25 ± 0.68 μg/ml*, P* = 0.048).

### Multiple regression analysis to identify variables independently associated with SUA/sCr variations

We performed a multiple regression analysis (Table 3) in order to identify variables independently associated with SUA/sCr variations.

**Table 3:** Multiple Regression Analysis Evaluating SUA/sCR as dependent variable

	Coefficient *β*	*P*
Multiple regression – Model 1*		
Age, years	−0.24	**0.0005**
Male sex	−0.29	** < 0.0001**
Smoking habit	0.003	0.99
BMI, kg/m^2^	0.13	0.060
SBP, mmHg	0.01	0.60
SGLT2i	**−**0.24	**0.0004**
Multiple regression – Model 2^†^		
Age, years	−0.25	**0.0002**
Male sex	−0.28	**0.0002**
SGLT2i	−0.24	**0.0006**
HbA1c, %	0.06	0.40
UACR > 30 mg/g	−0.07	0.27
LDL cholesterol, mg/dl	0.07	0.29
HDL cholesterol, mg/dl	0.004	0.96
Multiple regression – Model 3^‡^		
Age, years	−0.30	**0.0002**
Male sex	−0.38	** < 0.0001**
SGLT2i	−0.21	**0.0082**
u-B2M, ng/ml	−0.21	**0.0082**
u-Cys, ng/ml	−0.003	0.97
u-NGAL, ng/ml	−0.03	0.69
u-OPN, µg/ml	0.04	0.69
u-TFF3, µg/ml	−0.017	0.84

^*^Model 1 was adjusted for age, sex, smoking habit, BMI, SBP, and use of SGLT2i

^†^Model 2 was adjusted for HbA1c, UACR > 30 mg/g, LDL cholesterol, HDL cholesterol

^‡^Model 3 was adjusted for u-B2M, u-Cys, u-NGAL, u-OPN, u-TFF3

SUA/sCr: serum uric acid to serum creatinine ratio; BMI: body mass index; SBP: systolic blood pressure; SGLT2i: sodium-glucose cotransporter 2 inhibitors; HbA1c: glycated haemoglobin; UACR: Urinary Albumin to Creatine Ratio; LDL: low density lipoprotein; HDL: high density lipoprotein; u-B2M: urinary β2 microglobulin; u-Cys: urinary Cystatin C; u-NGAL: urinary Neutrophil Gelatinase-Associated Lipocalin; u-OPN: urinary osteopontin; u-TFF3: urinary TreFoil Factor 3. Bold: *P* < 0.05

In the first model SUA/sCr was associated with age (β = −0.24, *P* = 0.0005), male sex (β = −0.29, *P* < 0.0001), and therapy with SGLT2i (β = −0.24, *P* = 0.0004). In the second model, age (β = −0.25, *P* = 0.0002), male sex (β = −0.28, *P* = 0.0002), and therapy with SGLT2i (β =−0.24, *P* = 0.0006) remained significantly associated with PWV. Finally, in the third model SUA/sCr was independently and inversely correlated with age, male sex, therapy with SGLT2i, and u-B2M (β = −0.21, *P* = 0.0082). Sex did not show a significant interaction with the association between SUA/sCr and u-B2M (*P* for interaction = 0.74).

### Logistic regression analysis to identify variables independently associated with PWV > 10 m/s and the presence of carotid atherosclerotic plaque

As concern PWV > 10 m/s, within the first model it was associated with age (odds ratio [OR] 1.08, 95% confidence interval [CI] 1.02 – 1.14, *P* = 0.0052) and SBP (OR 1.04, 95% CI 1.01 – 1.06, *P* = 0.0060). Then, in the second model PWV > 10 m/s showed an independent association again with age (OR 1.09, 95% CI 1.03 – 1.15, *P* = 0.0040) and SBP (OR 1.03, 95% CI 1.01– 1.06, *P* = 0.019). Finally, in the third model, apart from age (OR 1.05, 95% CI 1.002 – 1.11, *P* = 0.043) and SBP (OR 1.04, 95% CI 1.01 – 1.06, *P* = 0.0053), PWV > 10 m/s was independently associated with SUA/sCr (OR 0.77, 95% CI 0.63 – 0.95, *P* = 0.014).

When considering the presence of carotid atherosclerotic plaques as dependent variables, in the first model, it was associated with age (OR 1.09, 95% CI 1.02 – 1.17, *P* = 0.012); while in the second model it was correlated again with age (OR 1.10, 95% CI 1.02 – 1.18, *P* = 0.012). Finally, in the third model age remained significantly associated with the presence of carotid atherosclerotic plaques (OR 1.07, 95% CI 1.05 – 1.18, *P* = 0.029), but SUA/sCr was also associated with it (OR 0.78, 95% CI 0.63 – 0.96, *P* = 0.020).

**Fig. 2:**
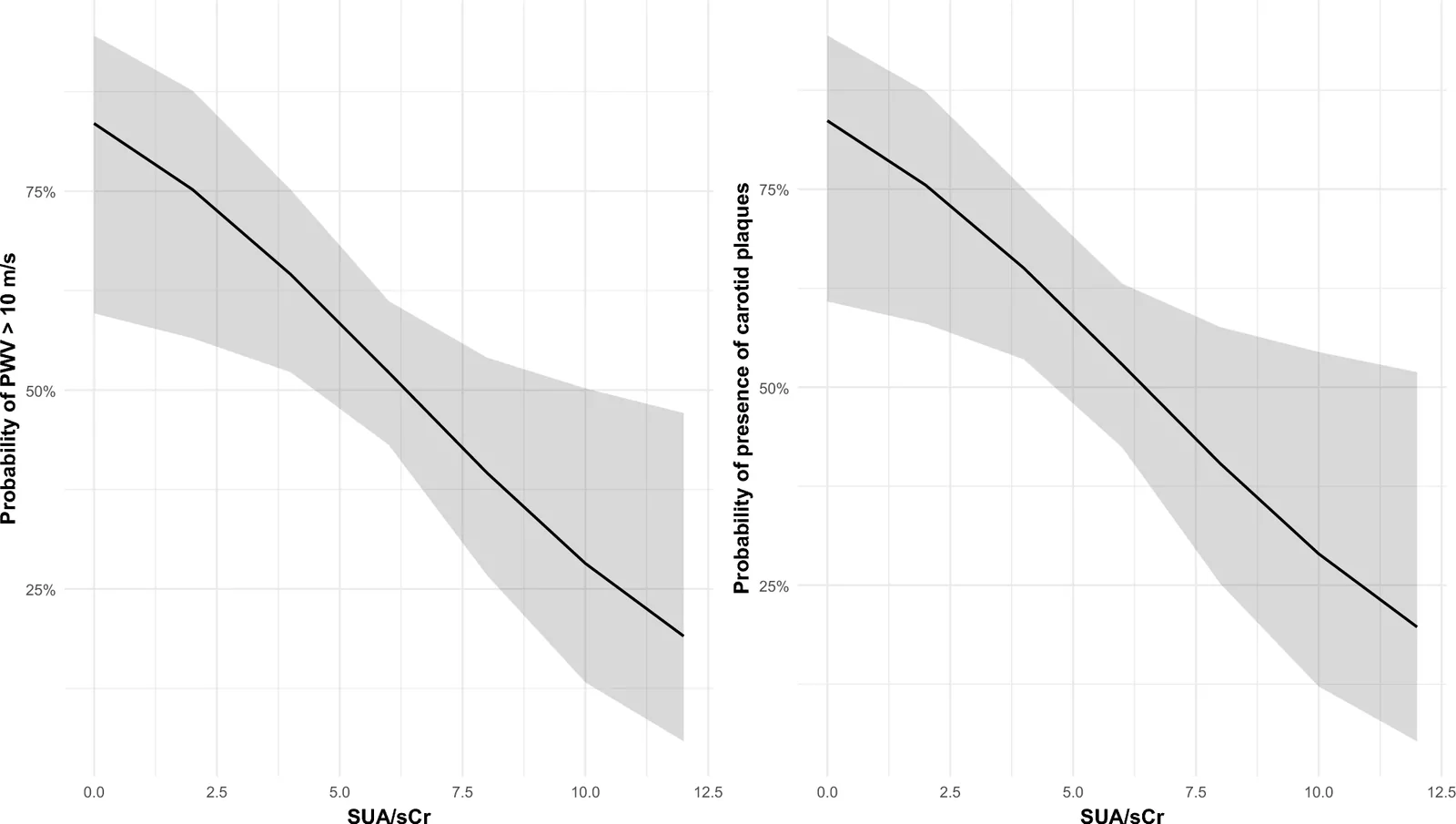
Predicted probability of PWV > 10 and carotid plaques according to SUA/sCr variations. The graphs show the predicted probability of PWV > 10 m/s (left panel) or having carotid atherosclerotic plaques (right panel) according to variations of serum uric acid to serum creatinine ratio (SUA/sCr) in multiple regression model 3, that includes age and SUA/sCr for carotid plaques and age, systolic blood pressure, and SUA/sCr for PWV > 10 m/s

Figure 2 represents the predicted probability within the third model of having PWV > 10 or carotid atherosclerotic plaques according to SUA/sCr variations.

### Multiple regression analysis to identify variables independently associated with RRI variations

In the first model of multiple regression analysis RRI showed an association with age (β = 0.44, *P* < 0.0001) and use of SGLT2i (β = 0.21, *P* = 0.036). Then, in the second model it remained associated with age (β = 0.45, *P* < 0.0001) and SGLT2i use (β = 0.21, *P* = 0.034) as well. Finally in the third model RRI was again associated with age (β = 0.39, *P* < 0.0001) and therapy with SGLT2i (β = 0.17, *P* = 0.017), but also with SUA/sCr (β = -0.13, *P* = 0.045).

## Discussion

In this study, we assessed subclinical vascular damage, as well as urinary biomarkers and ultrasound features of kidney damage in patients with DKD. In consideration of the increasing prevalence of NA-DKD, we particularly focused our attention on this phenotype.

We found an independent association between NA-DKD, and lower SUA/sCr, whereas no such relationship was observed in patients with A-DKD. The independent association between a lower SUA/sCr and NA-DKD phenotype was in line with the study by Mu et al. [12], which, identified SUA/sCr as independent predictor of NA-DKD and, additionally, this association was not observed in A-DKD patients. The difference between NA-DKD and A-DKD may be explained by the distinct patterns of kidney damage identified in these phenotypes, with NA-DKD characterized by prevalent vascular and tubulointerstitial damage [25, 26]. Interestingly, in our population, NA-DKD group showed increased u-TFF3, which is highly expressed in renal tubules and may play a role in epithelial regeneration [27]. Thus, elevated urinary levels of this peptide could indicate the presence of tubular injury in this phenotype of DKD.

Previous studies have demonstrated that several biomarkers may play a role for the early detection and monitoring of kidney injury in patients with NA-DKD [28, 29]. Nevertheless, their high cost, technical complexity and clinical availability limit their widespread use. Moreover, the relationship between vascular damage, renal impairment, and biomarkers could vary in the different stages of chronic kidney disease [30]. Accordingly, the need for simple, practical, clinical biomarkers for cardio-renal risk stratification in NA-DKD patients is still an unmet clinical need.

In DKD population, lower SUA/sCr was independently associated with subclinical vascular damage, as indicated by carotid atherosclerotic plaques and arterial stiffness (PWV > 10 m/s) and ultrasound-detected renal damage, as indicated by RRI.

Considering that both PWV and the presence of carotid plaques are predictive of cardiovascular events [31, 32], our results align with the evidence from the study by Tang et al. [13], which highlighted an inverse association between SUA/sCr and cardiovascular and all-cause mortality in adults with hypertension from the US National Health and Nutrition Examination Survey (NHANES) 1999–2018. In that study, participants with SUA/sCr ≤ 4 had increased hazard of all-cause anda cardiovascular mortality. In agreement with these results, Zhang et al. [33], in a large Chinese cohort of over 2,000 individuals, have shown that lower SUA/sCr was associated with a higher 1-year stroke recurrence rate after the first event.

The mechanisms linking a lower SUA/sCr with a worse vascular profile are not fully understood. Even though the cross-sectional design of this study does not allow to mechanistically explain this association, previous literature could suggest some involved pathways. A lower eGFR is widely recognized as a cardiovascular risk factor [34], however, more recently, tubular injury has emerged as possible independent risk factor for CVD and mortality [35, 36]. Thus, lower SUA/sCr could be associated with an impaired vascular profile as an expression of worse tubular function, when creatinine is comparably high.

In line with these considerations, we found a significant association between a lower SUA/sCr and u-B2M, a biomarker of renal tubular damage, that has been linked to proximal tubular damage in renal biopsies [37] and has been found elevated in other clinical conditions related to tubular injury [38]. Notably, urate secretion and reabsorption occur primarily in the proximal tubule. Thus, tubulointerstitial damage could affect these physiological processes, leading to a lower SUA/sCr [39]. The possible link between SUA/sCr and tubular damage is further supported by the independent correlation between this index and RRI in multiple regression models. Increased RRI, in fact, has been associated with renal tubulointerstitial damage in different clinical settings. Particularly, a RRI > 0.65 showed a specificity of 100% for biopsy confirmed tubulointerstitial injury in patients with glomerular diseases [40] and RRI allowed the early identification of both normotensive and hypertensive patients with chronic tubular damage [41]. In addition, also insulin resistance, that is strictly bounded to diabetes, could affect urate metabolism influencing renal transporters activity [42].

Considering all this evidence, SUA/sCr may be interpreted in a context-dependent manner, reflecting possible tubulointerstitial dysfunction and systemic vascular damage in the context of the cardio-renal-metabolic continuum, underscoring the close interplay between vascular and renal function [43, 44].

This study presents some strengths and limitations. As concern strengths, we obtained a complete cardiovascular and renal profile of different DKD phenotypes, which is still an underexplored field. In addition, we used well validated measurements, such as PWV, the presence of atherosclerotic plaques, and RRI. Furthermore, a comprehensive panel of renal injury biomarkers has been considered.

On the other hand, this was a cross-sectional, single-centre study, thus a longitudinal causal relationship cannot be established, and the generalizability of our findings may be limited. In addition, even if we considered sex as confounder in all the performed analyses, the higher prevalence of male sex could have conducted to residual confounding. The use of allopurinol or febuxostat were exclusion criteria and SGLT2i, which can exert a direct effect on uric acid excretion, have been included as confounders in the statistical analyses. Nevertheless, residual confounding due to indirect effects of other drugs on uric acid metabolism cannot be fully excluded. Data about insulin resistance and body composition was not available and for this reason it is not possible to evaluate their role in influencing uric acid metabolism and vascular parameters in this population. The lack of diet assessment could introduce residual confounding due to the role of diet in influencing uric acid levels. However, SUA/sCr does not represent a mere biochemical value, but it represents a more complex clinical phenotype that encompass both uric acid metabolism and kidney function.

Therefore, future studies, with a longitudinal design, could clarify the predictive value of SUA/sCr for DKD incidence and progression, as well as with cardiovascular events. Furthermore, the integration of SUA/sCr with metabolic, inflammatory and lifestyle markers as well as other vascular marker such as ankle-brachial index and the study of dietary and therapeutic interventions modifying SUA/sCr trajectories in NA-DKD could provide a new instrument for clinical risk stratification and individualized therapy.

In conclusion, low SUA/sCr was associated with early vascular damage and markers of tubular injury in patients with NA-DKD but not with A-DKD. These findings, if further confirmed by future studies, may suggest the possibility of evaluating SUA/sCr as candidate simple and cost-effective biomarker to improve risk assessment in people with diabetes and DKD.

## Data Availability

The dataset used and/or analysed during the current study is available from the corresponding author on reasonable request.
